# Oncoplastic Breast Surgery Taking “Center” Stage—Managing Centrally Located Breast Tumors in Indian Cohort

**DOI:** 10.3390/jcm15187076

**Published:** 2026-09-12

**Authors:** Chaitanyanand B. Koppiker, Aijaz Ul Noor, Rupa Mishra, Vaibhav Jain, Priya Sivadasan, Sneha Bhandari, Namrata Athavale, Vishesha Lulla, Sanika Limaye, Mugdha Pai, Chetan Deshmukh, Mansi Munshi, Beenu Varghese, Upendra Dhar, Sneha Joshi

**Affiliations:** 1 Prashanti Cancer Care Mission (PCCM), Centre for Translational Cancer Research, Pune 411048, Indiarupamishra@prashanticancercare.org (R.M.);; 2 Orchids Breast Health Centre, in Collaboration with PCCM and Jehangir Hospital, Pune 411001, India

**Keywords:** centrally located breast tumors, LMIC, oncoplastic technique, surgical audit

## Abstract

**Background:** Centrally located breast tumors (CLBTs) present unique challenges in surgical management, requiring a delicate balance between oncological outcomes and cosmetic preservation. Oncoplastic techniques have emerged as a promising solution, allowing larger excisions while maintaining cosmetic appearance and function. This study aims to evaluate the surgical management of CLBT using oncoplastic breast surgery (OBS) treated at a single surgeon center in India. **Methods:** Among 138 patients with CLBT who underwent OBS, diagnosis and staging adhered to NCCN guidelines, with treatment decisions made via a multidisciplinary tumor board and patient counseling. Intraoperative ultrasound, specimen mammography, and frozen-section evaluation guided resection margins and axillary staging. **Results:** The median patient age was 52.7 years. Seventy-three percent of patients had unifocal disease, with 67% presenting at the cT2–cT3 stage, and 50% being clinically node-positive. Level 1 OBS was used in 21.73% of cases, Level 2 in 42.75%, and Level 3 in 35%, with 28% qualifying as extreme oncoplasty. Post-surgical complications were observed in 13% cases. Cosmetic outcomes were good to excellent in 94% of cases. At a median follow-up of 57 months, overall survival (OS) was 91.93%, and disease-free survival (DFS) was 88.17%. The BREAST-Q PROM response rate was 94%, with high satisfaction across all domains. **Conclusions:** This study highlights the safety and efficacy of OBS in CLBT. Key factors such as multidisciplinary management, meticulous surgical planning, and rigorous intraoperative rad-path assessment contributed to its success. These findings highlight the potential of OBS to optimize outcomes and enhance quality of life, even in difficult cases and resource-limited settings.

## 1. Introduction

Oncoplastic breast surgery (OBS), which integrates the principles of oncologic and plastic surgery, has become increasingly prevalent, particularly in Western countries [1]. This technique involves meticulous planning of excision, breast gland reshaping, and repositioning of the nipple–areola complex (NAC) to achieve improved esthetic outcomes and breast symmetry [2]. OBS, including partial mastectomy with breast reduction, is increasingly being adopted as a standard surgical approach for early-stage breast cancer and, in selected cases, for locally advanced and large operable breast cancer [3,4].

Centrally located breast tumors (CLBTs) are defined as tumors that are completely or partially located in the projection of the areola up to the chest wall and/or within 2 cm around the areola [5]. Representing approximately 5–20% of cases, CLBTs pose significant surgical challenges due to higher rates of axillary lymph node metastasis, increased likelihood of positive margins and NAC involvement, less satisfactory cosmetic outcomes, and a higher risk of local recurrence [6,7,8,9]. Historically, mastectomy or radical mastectomy constituted the standard treatment for these tumors, due to concerns over local control and risk of multicentricity, rendering breast-conservative surgery relatively contraindicated. However, advances in surgical techniques, particularly the development of OBS, have provided an alternative option for selected patients [10,11]. Despite these advancements, the optimal surgical management for CLBTs remains a subject of ongoing debate, reflecting the complexity of balancing oncologic principles with esthetic outcomes and local control [12,13,14].

Current evidence regarding the application of OBS in CLBT remains limited and is largely focused on postoperative outcomes and oncological safety [15,16,17,18]. Existing data are constrained by several significant limitations: study design, small sample sizes, lack of uniformity in postoperative radiotherapy (RT) protocols, variability in reported postoperative outcomes, and inadequate follow-up durations. Collectively, these limitations hinder the ability to draw robust conclusions regarding the efficacy and safety of OBS in patients with CLBTs. Further research is required to evaluate the oncological outcomes of OBS in CLBT. Studies such as ours are crucial to accurately evaluate the outcomes, efficacy, and overall benefits of OBS in CLBT patients.

In low- and middle-income countries (LMICs) such as India, where mastectomy is often favored over breast conservation, this study’s center is dedicated to building evidence and stimulating a shift in perspective. The aim is to demonstrate that breast conservation is not only feasible but also beneficial, even in challenging clinical scenarios. Through meticulous patient work-up, comprehensive record-keeping, thorough diagnosis, multidisciplinary team involvement, and stringent radiological–pathological analysis, the center has established a strong breast oncoplastic practice that significantly enhances the quality of life for its patients. This study represents a 10-year audit of all patients presenting with CLBTs at the center. Given the unique challenges posed by CLBTs, the center has consistently employed OBS techniques to achieve optimal oncological and esthetic outcomes. Despite operating in a resource-constrained environment, the focus on advanced planning, collaboration, and patient-centered care has allowed the center to integrate OBS into routine practice.

Given the limited published data on OBS for CLBT from LMICs, this retrospective observational study evaluates the clinicopathological profile, postoperative outcomes, oncological safety, overall survival (OS), and disease-free survival (DFS) of patients treated using OBS techniques. These findings aim to provide breast onco-surgeons with evidence-based insights to support the adoption of OBS techniques, ultimately improving the management and outcomes of breast cancers even for difficult quadrants.

## 2. Methods

### 2.1. Study Design

In this retrospective, single-institutional, observational study, the medical records of breast cancer (BC) patients who underwent surgical treatment for CLBT at Orchids Breast Health Clinic (OBHC), Pune, between 2013 and 2023 were retrospectively reviewed. Informed consent was obtained from all patients for collection and use of clinical data related to disease management and routine follow-up visits. Collected data included demographic information, medical history, clinicopathological characteristics, surgical notes, postoperative evaluations, chemo-radiation treatment plans, and follow-up details.

### 2.2. Clinical Management

A triple assessment comprising clinical examination, appropriate breast imaging, and image-guided core needle biopsy was routinely performed to establish the diagnosis. Patients with histologically confirmed breast cancer underwent breast surgery at an affiliated network hospital. Following clinical staging, patients were selected for neoadjuvant chemotherapy/neoadjuvant hormonal therapy (NACT/NAHT) and adjuvant treatment based on decisions made by the multidisciplinary team (MDT), in accordance with the unit’s protocol and suggested global treatment, i.e., National Comprehensive Cancer Network (NCCN) guidelines. The choice of surgical intervention was determined based on tumor staging and patient characteristics. All patients in the study cohort underwent breast conservation surgery (BCS) with either Level 1, Level 2 or Level 3 OBS techniques [19].

### 2.3. Surgical Procedure

At this study’s center, various surgical procedures have been adopted for the management of CLBTs (1) Grisotti flap technique, (2) therapeutic mammoplasty, (3) perforator flaps.

#### 2.3.1. Surgical Procedure for Grisotti Flap

In this surgical procedure, the NAC was first demarcated, followed by marking another small circle just below the NAC and also the inframammary sulcus. The medial and lateral borders of the flap were then drawn extending from the medial and lateral margins of the areola down to the inframammary fold and converging distally to give a comma-shaped appearance. The flap was subsequently de-epithelialized, except for a circular area close to the defect. An incision was made along the medial and inferior borders of the flap down to the fascia. The flap was then undermined from the fascia laterally that so it can be rotated upwards and advanced superiorly to fill the defect.

**Figure 1:**
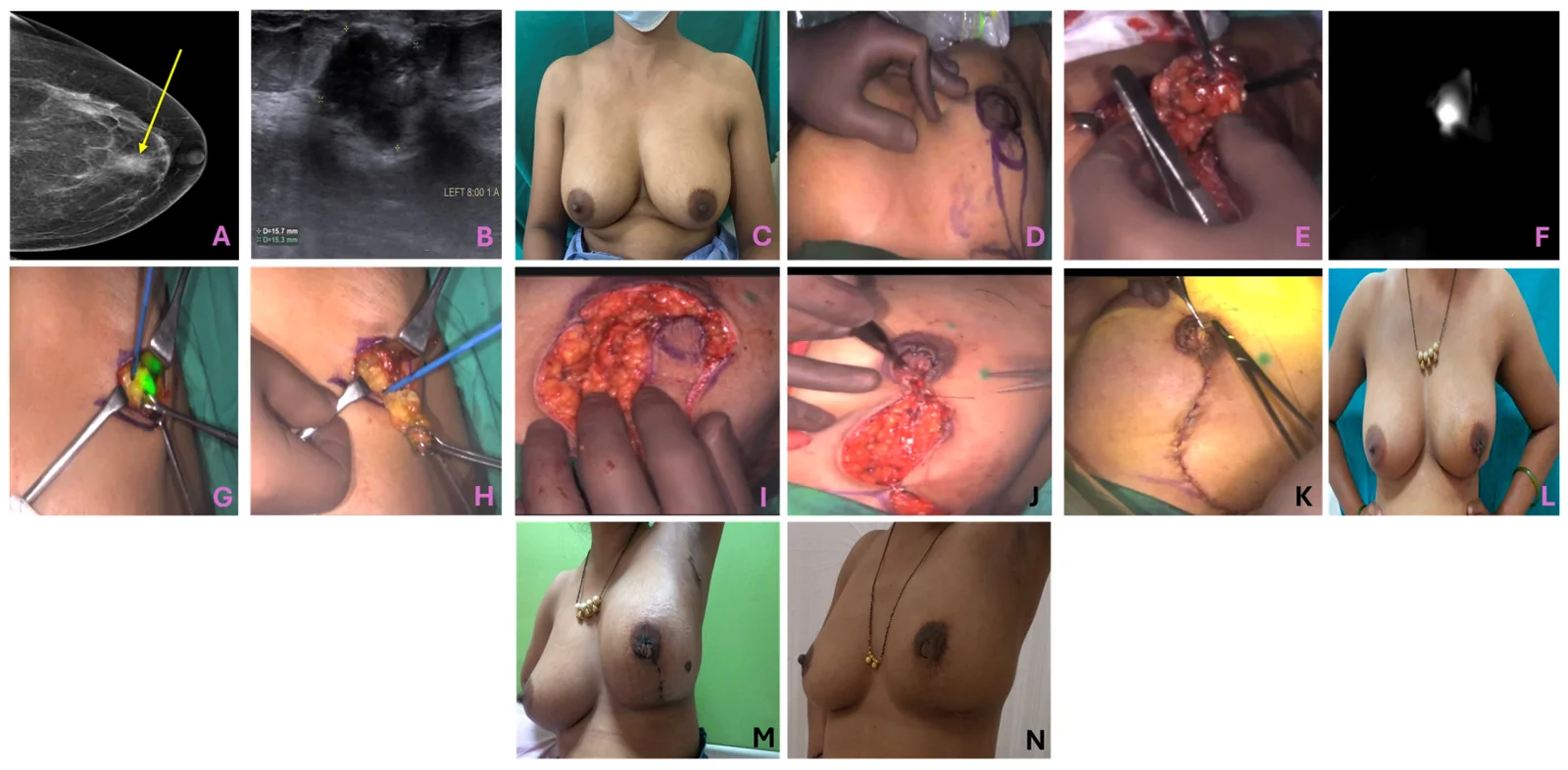
Case 1: Modified Grisotti—(**A**) tumor seen on 3D Tomo CC image; (**B**) USG image; (**C**) preop image; (**D**) preop marking; (**E**) tumor excised; (**F**) sentinel node detected through ICG dye; (**G**,**H**) sentinel node picked up; (**I**) Grisotti flap raised; (**J**,**K**) wound sutured; (**L**,**M**) postop; (**N**) postop after 8 months.

The NAC and tumor were excised en bloc during a central quadrantectomy, together with a column of tissue extending to the pectoral fascia. The tumor margins were indicated for intraoperative frozen-section investigation. The placement of titanium clips was planned for adjuvant radiation. The medial and inferior margins of the flap were then incised down to the pectoral fascia followed by the wide mobilization of the flap from the underlying pectoral fascia. The flap was subsequently advanced and rotated to fill the defect, and the wounds were closed with complete suturing. In selected cases, a second incision was made in the axillary fold to dissect the axillary lymph nodes [20]. The clinical presentation and surgical technique are described in Case 1, Video S1, and Figure 1.

#### 2.3.2. Surgical Procedure for Therapeutic Mammoplasty (TM)

**Figure 2:**
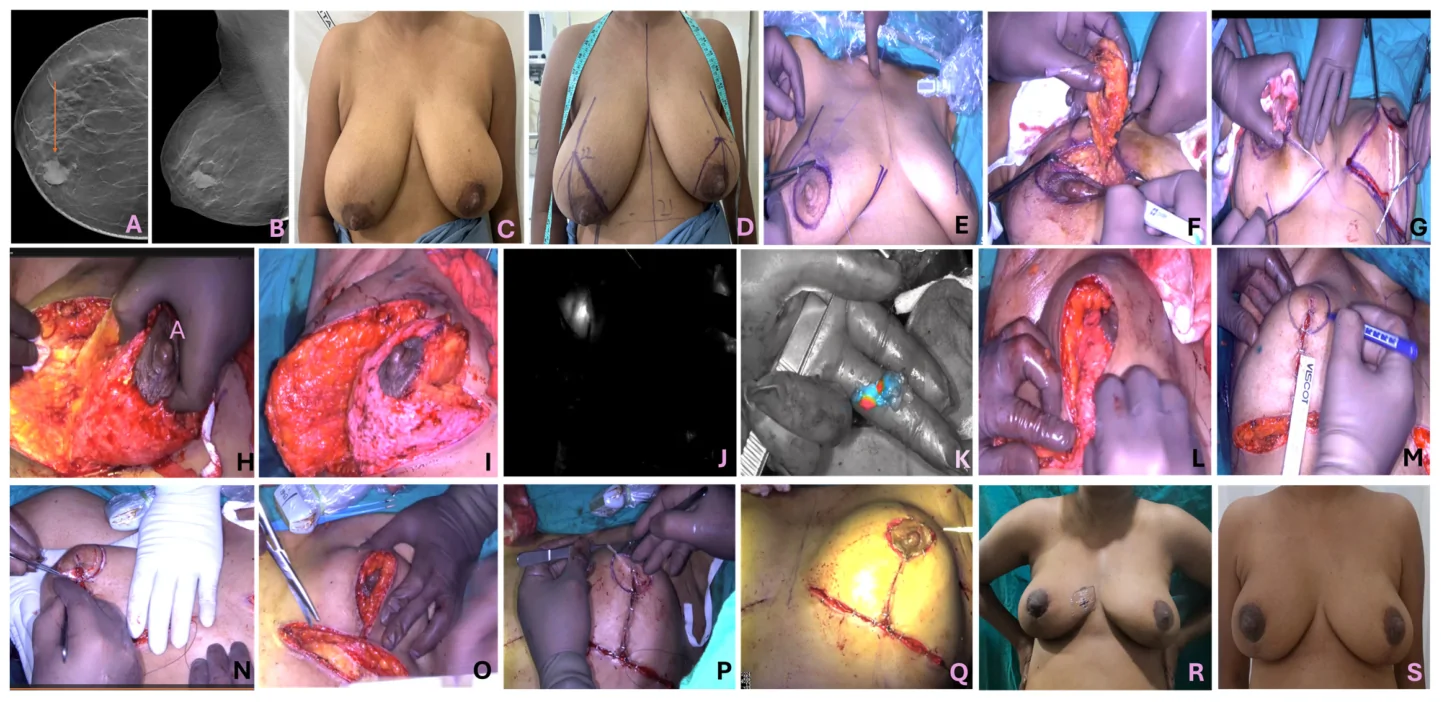
Case 2: Level 2—(**A**,**B**) tumor location on Mammogram—CC and MLO; (**C**–**E**) preop without and with marking; (**F**) tumor excised; (**G**) incision along Wise pattern marking; (**H**,**I**) inferior pedicle; (**J**,**K**) sentinel node located with ICG dye and picked up; (**L**) pillars closed; (**M**) marking for NAC and distance from inframammary fold measured; (**N**) de-epithelization done for reconstruction of NAC; (**O**–**Q**) contralateral symmetrization with NAC reconstruction; (**R**,**S**) postop after radiation therapy and postop after one year.

Therapeutic mammoplasty (TM) is an oncoplastic surgical technique that combines oncological safety with favorable esthetic outcomes for selected breast tumors in moderate-to-large breasts. Following Wise pattern incisions, a central wedge of breast tissue containing both the nipple and tumor is removed. The margin is usually very wide around the tumor. An inferior pedicle carrying the parenchyma and overlying skin is then mobilized and advanced into the central defect. Since the nipple is not reconstructed, the use of parenchymal or nipple–areola complex pedicles is avoided. The pillars are then simply sutured together and the skin is closed. In selected patients, the lateral dissection is extended into the axilla to facilitate axillary management, including sentinel lymph node biopsy (SLNB) or axillary lymph node dissection (ALND) as indicated. No separate axillary incision is made [21,22,23,24,25,26]. The clinical presentation and surgical technique is described in Case 2, Video S2, Figure 2.

#### 2.3.3. Surgical Procedure for ICAP (MICAP, AICAP, LICAP) and LTAP Flaps

Flap selection was based on tumor location and defect size, using single or combined perforators (LTAP, LICAP, MICAP, AICAP) to optimize volume and minimize scarring [27,28,29,30]. Preoperative Doppler confirmed perforator presence, and patients were counseled on alternatives if needed.

**Figure 3:**
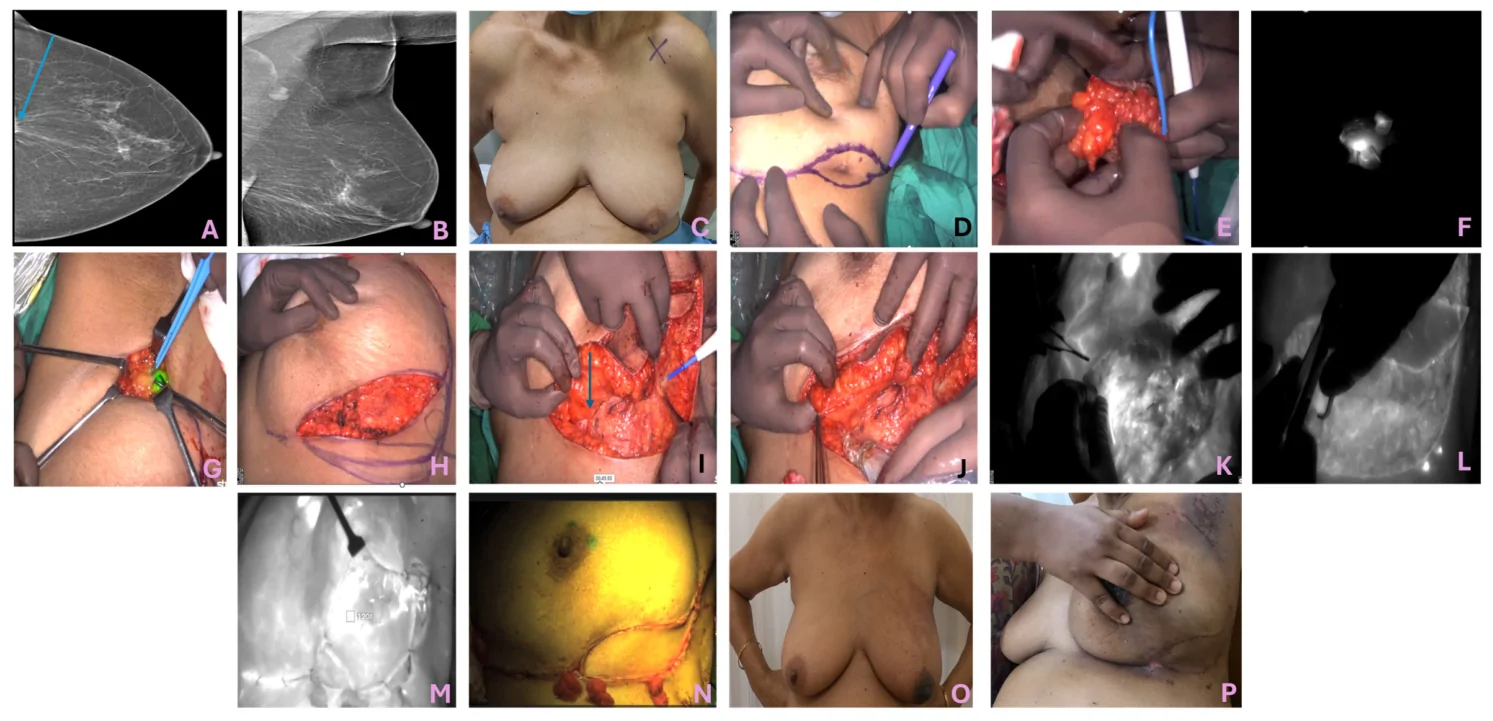
Case 3: Level 3—(**A**) location of tumor CC image; (**B**) MLO view; (**C**) preop image; (**D**) marking of tumor area; (**E**) tumor excised; (**F**) sentinel node detected through ICG dye; (**G**) sentinel node excised; (**H**) flap marking done; (**I**) perforator vessel located; (**J**) pulsation checked through Doppler; (**K**) vascularity of the flap checked; (**L**) vessel clamped; (**M**) perfusion score of the flap assessed; (**N**) flap closure, wound closed; (**O**,**P**) postop images.

On the day of surgery, the perforators were re-confirmed and marked. SLNB was performed using indocyanine green (ICG) dye, and flaps were raised as planned. Tumor excision and axillary dissection was performed adhering to oncological safety, with the ICG dye assessing the flap vascularity as well as the perfusion assessment before the final placement. Detailed procedural steps are described in this study’s authors’ previous study on perforator flaps [31]. The clinical presentation and surgical technique are described in Case 3, Video S3, and Figure 3.

### 2.4. Post-Surgery Outcome Measures

#### Cosmetic Assessment

Cosmetic outcomes following breast-conserving surgery were evaluated between 3 and 6 months after surgery. Assessment was performed using the Harvard cosmetic scale, a four-point physician-rated scale that categorizes cosmetic outcome as excellent, good, fair, or poor. For analytical purposes, excellent and good outcomes were grouped as good–excellent cosmetic outcomes [32].

Patient satisfaction and quality of life following breast conservation were assessed using the standardized BREAST-Q BCT questionnaire 12 months post-surgery [33]. The BREAST-Q BCT module comprises multiple domains assessing various aspects of patient-reported outcomes. Each domain was scored on a 0- to 100-point scale with higher scores indicating greater satisfaction.

### 2.5. Radiation Therapy (RT)

Adjuvant radiotherapy was delivered to the whole breast using a moderately hypo-fractionated regimen of 40 Gy in 15 fractions over 3 weeks (2.67 Gy per fraction), using tangentially placed field-in-field intensity-modulated radiotherapy (FiF-IMRT) with daily image guidance (IGRT). Tumor bed boost was either simultaneously integrated at 48 Gy in 15 fractions or as a sequential boost of 12.5 Gy in 5 fractions. Regional nodal irradiation encompassing the supraclavicular fossa (SCF) was included in all node-positive patients. Targeted axillary irradiation was delivered selectively to patients with SLNB-confirmed nodal involvement in whom ALND was not performed, or in whom residual axillary disease was suspected after ALND based on histopathology or intraoperative findings. Therapeutic internal mammary chain (IMC) irradiation was delivered selectively to patients with imaging-confirmed IMC nodal involvement on preoperative or pre-chemotherapy staging imaging.

### 2.6. Statistical Analysis

Descriptive statistics and other analyses were performed using Microsoft Power BI (Version 2.152.882.0, 64-bit, March 2026). Kaplan–Meier survival analyses were performed using Python 3 (Version 3.12.4) within a Jupyter Notebook (Version 7.0.8) environment. Overall survival (OS) was calculated as all-cause since in many cases it was difficult to ascertain if death was due to the breast disease or other unrelated causes. The overall survival interval was taken as the period between date of diagnosis and death. Disease-free survival (DFS) was defined as the interval between the date of surgery and the date of first documented recurrence. Patients who did not experience the event of interest were censored at the date of their last clinical follow-up.

## 3. Results

### 3.1. Patient Demographics

Of the 171 (2013–2023) breast disease patients who presented with CLBT at this study’s center, 80.7% were surgically managed with BCS and 19.2% underwent mastectomy. In the current study, the data are presented for the 138 patients who underwent BCS, of which 137 were malignant breast cancer cases and one patient presented with a benign condition. Among the presented BCS cohort, Level I/simple oncoplastic closures were performed for 21.73% cases, Level II for 42.75% and Level III for 35% cases.

Patient demographic details and tumor characteristics for the cohort are summarized in Table 1. Median age was 52.7 years (range: 22–77). Forty-eight-point-five percent (67/138) of the patients in the cohort had comorbidities such as diabetes and hypertension, thus making them ineligible for mastectomy with immediate reconstruction.

**Figure 4:**
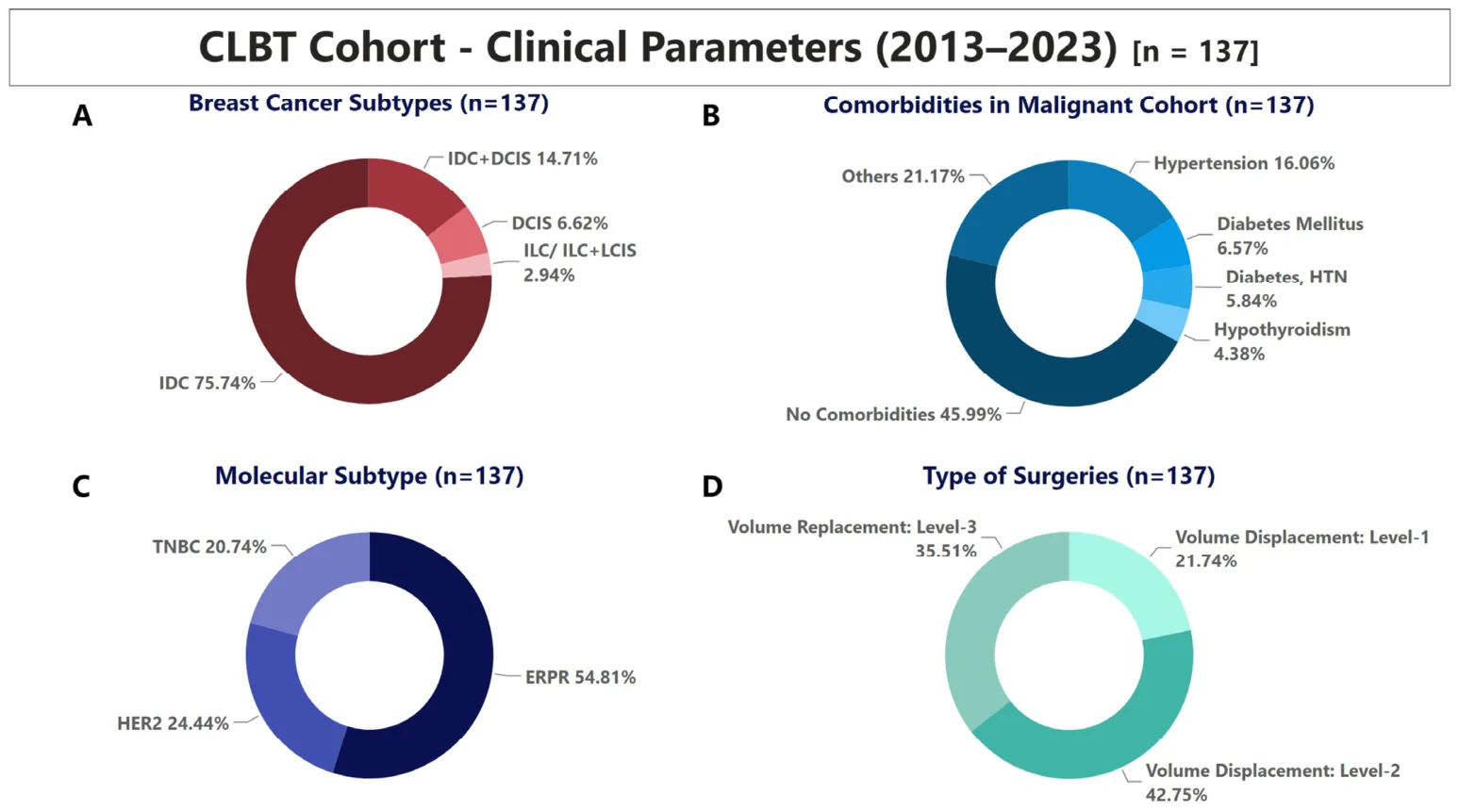
This figure represents the clinical parameters of the cohort (2013–2023): (**A**) breast cancer subtypes, (**B**) comorbidities in breast cancer patients, (**C**) molecular subtype distribution, (**D**) types of surgeries.

Sixty-seven percent of breast cancer patients in the cohort presented at cT2-cT3 stage and almost 50% of the cohort were clinically node-positive. Table 2 summarizes the detailed description of the clinicopathological parameters of the breast cancer patients in the cohort. Table 3 and Table 4 detail the distribution of the surgical techniques utilized for management of CLBT. Of the 137 breast cancer patients, 54 (39.4%) patients received NAST, 130 (95%) ACT, and 135 (98.5%) RT. Figure 4 summarizes the clinical parameters of the breast cancer patients in the cohort.

### 3.2. Neoadjuvant Chemotherapy (NACT/NAHT)

Of the 137 breast cancer patients, 54 (39.4%) received NAST. Among the 54 patients who received NAST, pathological complete response (pCR) was observed in 17% (9/54) of patients.

### 3.3. Surgical Outcomes

#### 3.3.1. Surgical Margins and Nodal Clearance

Clear margins were achieved in all the malignant cases. Margins were declared “clear” if no ink on the tumor was observed in cases of invasive carcinoma and a minimum margin width of 2 mm was “clear” of the ink on the tumor in cases of pure DCIS. Of the 137 breast cancer cases, axillary management was performed for 125 patients, of which only SLNB was done for 64 (51.2%), direct ALND for 36 (28.8%) and 25 (20%) had both SLNB and ALND.

Within the current cohort, the extreme oncoplasty [22,34,35] subgroup comprising 38 breast cancer cases, who had multifocal/multicentric disease, extensive DCIS or node-negative tumors larger than 5 cm. On descriptive review, outcomes among patients undergoing extreme oncoplasty appeared broadly comparable to those undergoing non-extreme oncoplastic procedures, with no clear excess in positive margins, postoperative complications, or local recurrence observed in the extreme oncoplasty subgroup. However, due to the smaller sample size, no statistical comparisons and conclusions could be derived.

#### 3.3.2. Postoperative Complications

**Figure 5:**
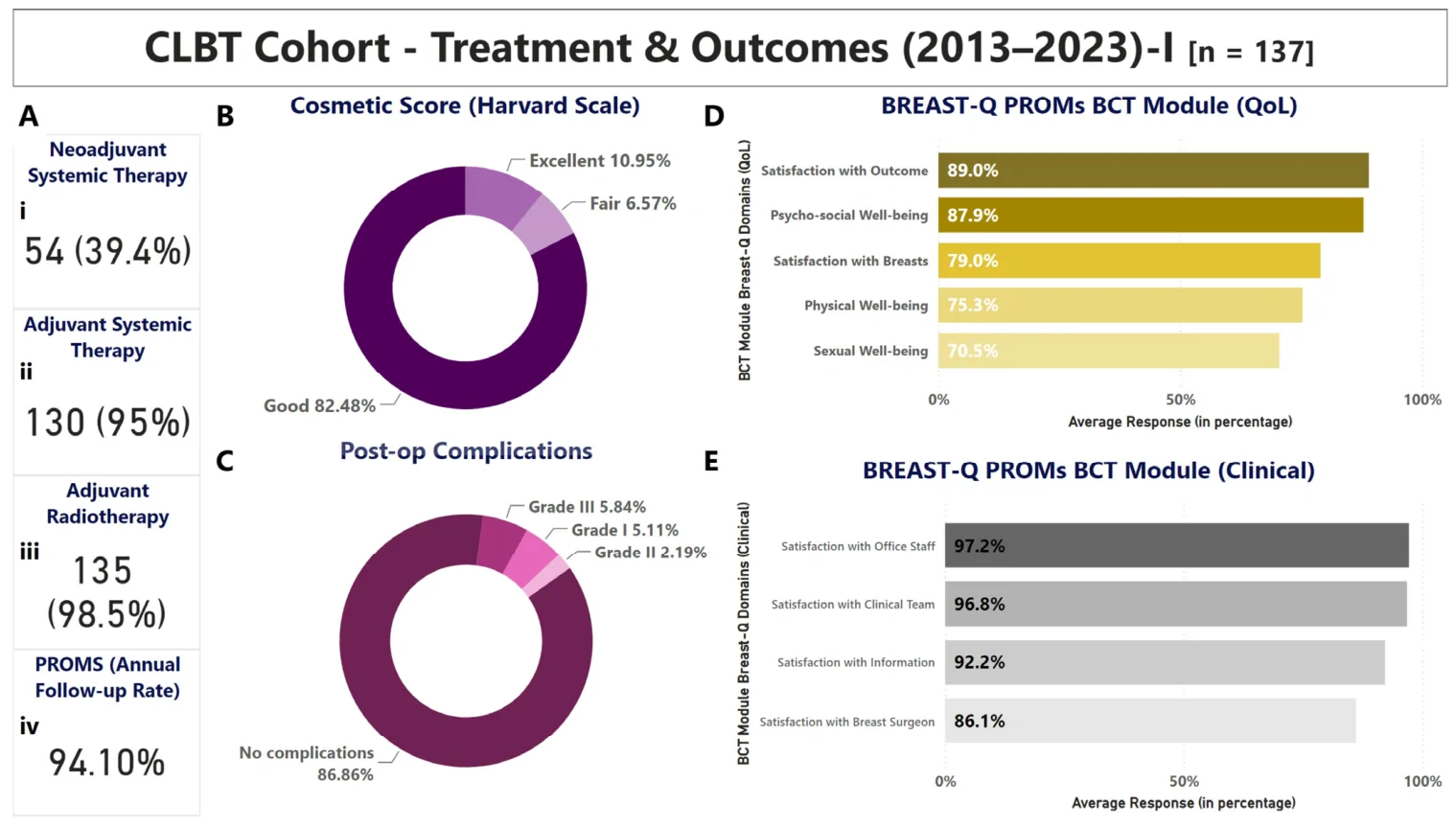
This figure presents outcomes and follow-up data from study cohort (2013–2023). (**A**). Neo and adjuvant therapy and follow-up statistics: (**Ai**). Neoadjuvant systemic therapy. (**Aii**). Adjuvant systemic therapy. (**Aiii**). Adjuvant radiotherapy. (**Aiv**). PROMs (patient-reported outcome measures). (**B**). Cosmetic scores (Harvard scale). (**C**). Postop complications (**D**). Patient-reported outcome (PROM) scores (QoL). (**E**). Patient-reported outcome (PROM) scores (clinical).

Postoperative complications were classified based on grades as per the Clavien–Dindo classification adapted for breast cancer [36]. A total of 18/138 (13%) (Table 5, Figure 5C) cases of complications were observed. All complications were treated conservatively in the outpatient setting.

### 3.4. Adjuvant Radiotherapy

Of the 137 breast cancer patients included in the current study cohort, 135 (98.5%) patients underwent RT as clinically indicated. Among those who received RT, 80 (59%) patients did not have any adverse reactions to the RT, 56 developed adverse reactions with 52 (38.5%) developing Grade I–II reactions, and three (2.5%) developing Grade III–IV reactions.

### 3.5. Survival Outcomes

**Figure 6:**
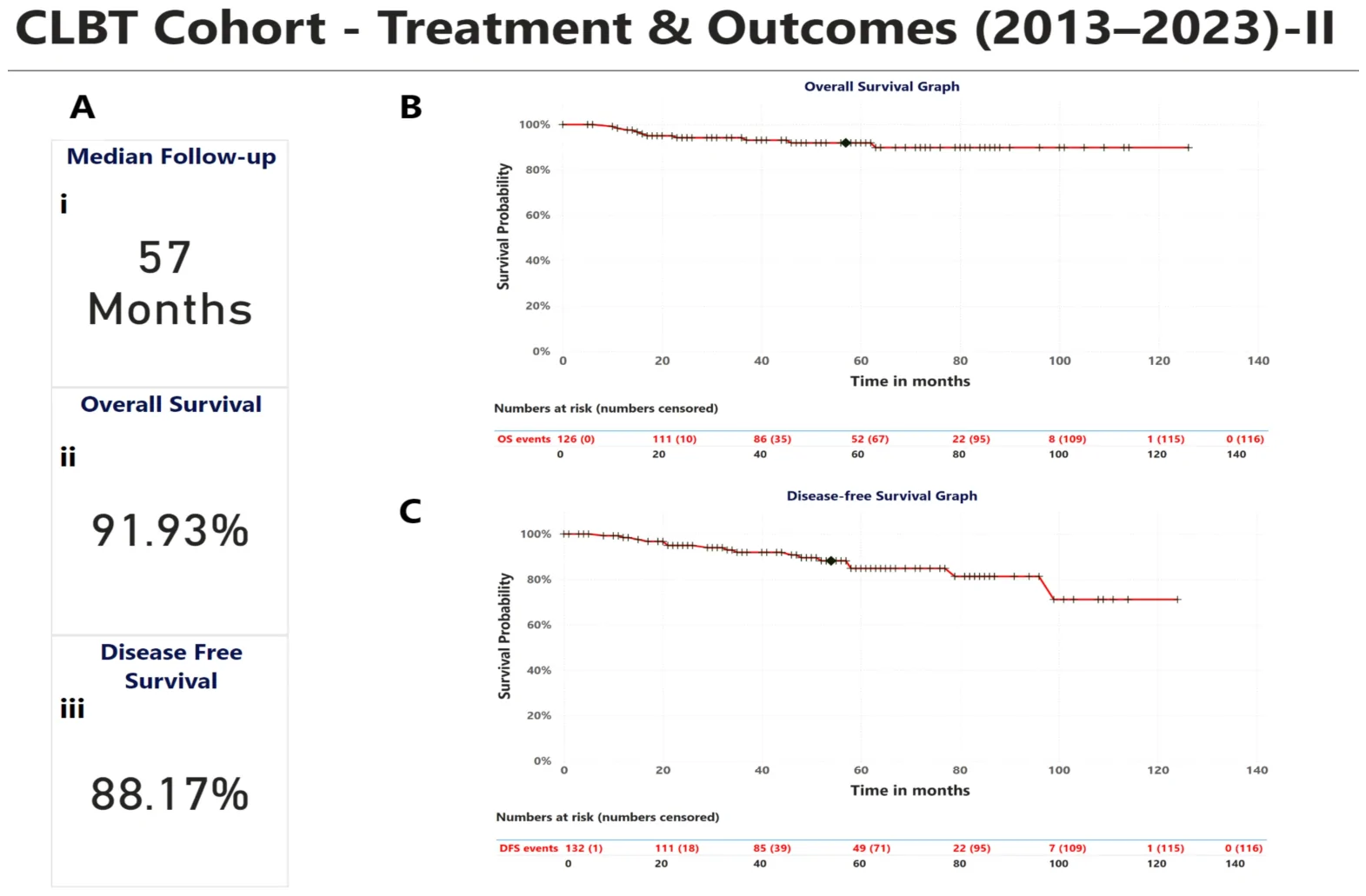
Kaplan–Meier survival plots of disease-free and overall survival. (**Ai**) Median follow-up; (**Aii**) overall survival at median follow-up; (**Aiii**) disease-free survival at median follow-up. (**B**) In overall survival, the approximate date of death due to any cause was taken as an event. Time is the period in months between surgery and the approximate cause of death and is shown in months. (**C**) In disease-free survival, local and distant recurrences and metastasis are taken as events. Time is the time period in months between surgery and the known date of evidence of recurrence. Vertical bars indicate censored patients in both plots.

The median follow-up was 57 months, with five breast cancer patients lost to follow-up. Within the cohort of 137, a total of 16 cases (11.67%) of recurrence were observed with nine local (6.56%) and seven (5.10%) distant recurrences over the complete follow-up available at the time of this report. At the median follow-up, the OS was found to be 91.93% (95% CI: 85.0–95.8%). In addition, the DFS at median follow-up was 88.17% (95% CI: 79.9–93.2%). KM plots of OS and DFS are shown in Figure 6.

### 3.6. Cosmetic Score Analysis

The cosmetic scores for the 137 breast cancer patients undergoing BCS were assessed by external breast and plastic surgeons between 3 and 6 months post-surgery. Good–excellent cosmetic scores were observed for over 94.1% cases (129/137). Figure 5B shows the cosmetic scores as reported by the surgeons.

### 3.7. Patient-Reported Outcome Measures (PROMS)

PROM data were collected from the study participants after a minimum period of 12 months post-surgery using the BREAST-Q questionnaires. Out of 137 breast cancer patients, 129 (94.1%) responded to the questionnaire. High patient satisfaction scores were observed from the study cohort’s PROM data as visualized in Figure 2E and Figure 5D.

## 4. Discussion

The management of breast cancer based on tumor location has evolved significantly over time. Although CLBTs present greater surgical complexity and were traditionally managed with mastectomy, advances in surgical techniques, NAST and RT have significantly improved the feasibility of BCS for these tumors. Oncological outcomes following BCS have been reported to be comparable to those achieved with mastectomy, and these results have been further enhanced through the incorporation of OBS techniques [37,38,39,40].

CLBT account for approximately 11–26% of all breast cancer cases, presenting a unique challenge in terms of management and treatment approaches [6,8]. Historically, CLBTs have been associated with a less favorable prognosis compared with the tumors located in the lateral quadrants. This poorer prognosis has been attributed to the higher rates of axillary lymph node metastasis, increased likelihood of positive surgical margins and NAC involvement, less satisfactory cosmetic outcomes, and a higher risk of local recurrence. [6,7]. Additional concerns regarding local control failure and multicentricity have further complicated management decisions for these tumors [10,11].

Studies by Zucali et al. (1998) and Lohrisch et al. (2000) demonstrate a 20% increase in mortality and two-fold-higher risk of relapse for medially located tumors compared with the laterally located tumors [41,42]. This unfavorable prognosis led to recommendations for irradiation of IMC lymph nodes to reduce the risk of relapses [41,42]. Hammer et al. (2002) confirmed that patients with CLBTs experienced poorer survival and local control rates, while also supporting the potential benefit of IMC lymph node irradiation in improving clinical outcomes [43]. Gaffney et al. (2003) reported that CLBT adversely affected both breast cancer-specific survival (BCSS) and overall survival (OS), further reinforcing the rationale for IMC lymph node irradiation in management of these tumors [44]. Knauerhase et al. (2008) with a median follow-up of 94 months, identified CLBTs as a significant risk factor for breast cancer recurrence. The cumulative 10-year breast recurrence rate was reported as 22.5% ± 8.3% compared to 6.9% ± 2.3% for lateral breast tumors and CLBTs, respectively [45].

Collectively, the available evidence consistently associates CLBTs with poorer clinical outcomes, including increased risk of relapse, inferior local control, higher rates of breast recurrence, and adverse effects on both BCSS and OS. These findings support the consideration of IMC lymph node irradiation as a potential strategy to improve outcomes for patients with CLBT, although the role of this approach remains an area of ongoing debate and investigation.

The role of breast-conserving therapy (BCT), particularly for patients with CLBTs, has evolved substantially over time, reflecting broader advances in breast cancer management. Early studies and clinical trials involving mastectomy as the surgical option for CLBT management often restricted BCT for CLBT due to concerns about oncological safety and cosmetic outcomes. However, subsequent studies demonstrated that breast conservation could be both safe and effective in appropriately selected patients [13,46,47,48,49]. Further advances in OBS have improved esthetic outcomes, making BCT a viable option for a broader range of patients, including those with CLBTs [50,51,52,53].

The retrospective analysis of the NSABP trial by Fisher et al. reported an 11.1% incidence of pathological infiltration of the nipple, further underscoring concerns about the suitability of BCS in cases involving the nipple and/or NAC [38]. Historically, conservative surgery was contraindicated for CLBTs due to concerns regarding local control failure and tumor multicentricity [10,11]. These concerns may in part be explained by high rates of NAC involvement and poor esthetic outcomes [8,54,55,56,57]. Consequently, patients with CLBTs were often denied the opportunity for breast conservation, given the possibility of multifocality, multicentricity, direct invasion of the nipple–areolar complex (NAC), and esthetic revulsion arising from the possible removal of the NAC [58]. The failure of classical BCS techniques to adequately address these challenges led to the development and advancement of oncoplastic surgery, enabling wider excisions for BCS without compromising the natural shape of the breast (Clough improving). Several oncoplastic techniques are currently available for breast reconstruction following central quadrantectomy. The choice of the oncoplastic technique depends on tumor size, NAC involvement, the breast volume, and its ptotic degree [59,60,61,62,63].

Within the overall CLBT cohort evaluated in the present study, OBS was the predominant surgical approach, utilized in 80.7% of patients, whereas 19.2% underwent mastectomy. Among 171 patients with breast disease, 138 underwent OBS for CLBTs. The findings demonstrated minimal postoperative complications, favorable esthetic outcomes, and high patient-reported outcome measures. These results further support OBS as an excellent breast conservation strategy for centrally located defects, even in low-resource settings, consistent with the observations reported recently by Zhyghulin et al. [5].

The current study’s findings demonstrate the successful management of CLBTs through breast conservation and advanced OBS techniques, achieving high levels of patient satisfaction despite the unique challenges associated with practicing in a resource-constrained LMIC setting. By integrating oncoplastic principles into routine clinical practice, the current study’s center has substantially expanded the scope of breast conservation, even for complex cases involving difficult quadrants. Favorable cosmetic outcomes were achieved, with 11.59% of patients rated as having excellent outcomes and 81.88% rated as having good outcomes, validated by high patient-reported satisfaction scores (PROMs), highlighting the esthetic and psychological benefits of the oncoplastic approach.

Postoperative complications were infrequent (13%) and were predominantly managed conservatively, with rare Grade III complications, reinforcing that advanced oncoplastic techniques do not appear to increase the risk of severe complications. Oncological outcomes were robust, with a median follow-up of 57 months revealing low local recurrence (6.56%) and distant recurrence (5.10%) within the cohort of 137, alongside high OS (91.93%) and DFS rates (88.17%). These findings underscore the effectiveness of combining meticulous surgical techniques with appropriate adjuvant therapies to achieve excellent long-term oncological outcomes.

In LMICs, the management of breast cancer is fraught with unique challenges, many of which stem from systemic and socioeconomic barriers. Late detection and a high prevalence of locally advanced disease is common due to limited access to regular screening programs, lack of awareness, and delays in seeking medical care. Financial constraints further compound these issues, as many patients are uninsured and must bear the total out-of-pocket costs for treatment. This often results in incomplete or suboptimal care. The present cohort illustrates these challenges. Among 33 HER2+ cases, only 17 were able to afford trastuzumab (Herceptin), and only 12 completed the full course of therapy. Despite these constraints, five of these 12 patients (42%) achieved pCR, and the early HER2+ subgroup that received complete therapy demonstrated an impressive pCR rate of 60%. However, the overall pCR rate remained low, reflecting broader systemic inequities in access to comprehensive neoadjuvant treatment.

### Limitations of the Study

This study is based on a single surgeon cohort, resulting in a relatively limited sample size of 138 patients with CLBTs managed using OBS techniques.

In India, where mastectomy rates remain disproportionately high, this study’s center is actively working to promote breast conservation and OBS techniques through initiatives such as the ISOS program [64]. This program is designed to facilitate the implementation of the center’s surgical algorithm in other low-resource centers, making breast conservation a practical and accessible option even in resource-constrained settings.

Although all the procedures in the current study were performed at a single institution by the same operating team, ensuring consistency in surgical technique, external validation in diverse clinical settings is necessary to determine the generalizability of these findings. The limited utilization of BCS in India continues to represent a significant challenge. The current study center’s efforts are directed toward addressing this gap, but future studies adopting a multicentric approach with larger case numbers are essential to further establish the utility and outcomes of OBS in similar settings.

Another important limitation, particularly relevant to LMICs, is variability in access to systemic therapies. Particularly, financial constraints may limit access to trastuzumab for some HER2-positive patients, thus influencing treatment response rates and survival outcomes [65,66].

The median follow-up of the current cohort is 57 months. To strengthen the evidence base, the center will continue stringent long-term follow-up of this cohort to track oncological and patient-reported outcomes over time. Expanding research with large cohorts will be crucial to substantiating these initial observations and advancing breast conservation in India and similar low-resource settings.

## 5. Conclusions

Overall, the findings of the current study demonstrate the significant potential of OBS to achieve an effective balance between oncological efficacy and esthetic outcomes in the management of CLBTs. The favorable oncological outcomes, low postoperative complication rates, and high levels of patient satisfaction emphasize the benefit of OBS as a breast-conserving approach in this challenging subset of patients. However, variation in response to NAST and the aggressive nature of triple-negative breast cancer (TNBC) highlight the need for continued research and individual treatment strategies. This data support the continued evolution and refinement of oncoplastic techniques to enhance patient outcome and satisfaction in the management of CLBTs.

## Figures and Tables

**Table 1:** Demographics and tumor characteristics among study patients with OBS (N = 138).

Characteristics	Class Interval	Number	Percentage
Patient Age (Years)	≤35	5	3.6%
	36–50	60	43.4%
	≥51	73	52.9%
	Median Age	52.7	
Size of Breast	S	23	16.7%
	M	84	60.9%
	L	29	21.0%
	XL	1	0.7%
	NAV	1	0.7%
Ptosis	No Ptosis	12	8.7%
	Grade I—Mild	40	29.0%
	Grade II—Moderate	47	34.0%
	Grade III—Severe	38	27.5%
	NAV	1	0.7%
Focality	Unifocal	101	73.1%
	Multifocal/Multicentric	37	26.8%
Tumor Location	Periareolar	03	2.1%
	Retroareolar	73	52.9%
	Central Quadrant/ Deep Central Quadrant	62	45.0%
Tumor Biology Based on Biopsy	IDC	103	74.6%
	ILC/ILC + LCIS	4	3.0%
	IDC + DCIS	20	14.5%
	DCIS	9	6.5
	Benign	1	0.7%
	NAV	1	0.7%
Molecular Subtype	ER/PR	74	53.6%
	HER2	33	24.0%
	TNBC	28	20.3%
	Benign	1	0.7%
	NA	2	1.4%

**Table 2:** Clinicopathological features of breast cancer patients in the cohort.

Feature	Class	Total N = 137	
Focality	Unifocal	100	73%
	Multifocal/Multicentric	37	27%
In Unifocal Clinical Tumor Size (cT)	cT1	36	26.2%
	cT2	55	40.1%
	cT3	5	3.6%
In Multifocal Clinical Tumor Size (cT)	cT1	4	2.9%
	cT2	29	21.1%
	cT3	3	2.1%
DCIS	Tis	4	2.9%
Revised Surgery		2	1.4%
Tumor Grade	I	12	8.7%
	II	94	68.6%
	III	31	22.6%
	NA	20	-
Type of Tumor (Biopsy)	IDC	103	75%
	IDC + DCIS	20	14.5%
	ILC/ILC + LCIS	4	2.9%
	DCIS	9	6.5%
	NA	1	0.7%
Clinical Tumor Stage 83/137 (60.6%) Patients Underwent Upfront Surgery	Stage 0	5	3.6%
	Stage IA	24	17.5%
	Stage IB	0	-
	Stage IIA	32	23.3%
	Stage IIB	21	15.3%
	Stage IIIA	1	0.7%
	Stage IIIB	0	-
	Stage IIIC	0	-
	NA	0	-
Clinical Node Positivity (Upfront Surgery)	29 of 84 (34.5%) were node-positive		
Clinical Tumor Stage (Given NAST) 54/137 (40.14%) Patients Received NAST	Stage 0	0	-
	Stage IA	5	2.9%
	Stage IB	0	-
	Stage IIA	10	7.3%
	Stage IIB	26	18.9%
	Stage IIIA	12	8.7%
	Stage IIIB	0	-
	Stage IIIC	1	0.7%
	NA	0	0.7%
Clinical Node Positivity (NAST)	42 of 54 (77.7%) were node-positive		
Pathological Tumor Stage (Upfront Surgery)	Stage 0	2	
	Stage IA	24	
	Stage IB	1	
	Stage IIA	34	
	Stage IIB	19	
	Stage IIIA	2	
	Stage IIIB	0	
	Stage IIIC	1	
Pathological Tumor Stage (Post-NAST Surgery)	Stage 0 (pCR)	9	17% (9/54) No Residual Disease
	Stage IA	11	
	Stage IB	1	
	Stage IIA	11	
	Stage IIB	11	
	Stage IIIA	5	
	Stage IIIB	2	
	Stage IIIC	3	
	ypTx	1	

**Table 3:** OBS technique for CLBTs.

OBS Technique (N = 138)	Number	Percentage
Simple Oncoplastic Closure/Level I	30	21.73%
Level II OBS (Therapeutic Mammoplasty)	59	42.75%
Level III OBS (Perforator Flaps)	49	35.5%

**Table 4:** Types of Level I OBS techniques.

Level I OBS Technique	Number
Grisotti Flap	11
Round Block	8
Simple Oncoplastic Closure	11

**Table 5:** Postop complications in the cohort as per the Clavien–Dindo classification.

Characteristics	Complications
Grade I (Seroma/hematoma not requiring drainage; minor skin necrosis; fat necrosis; delayed wound healing)	7
Grade II (wound infection)	3
Grade IIIa (Seroma/hematoma were drained under USG guidance; lymphedema; nipple necrosis; skin necrosis undergoing debridement)	1
Grade IIIb (Seroma/hematoma drained under general anesthesia—major skin necrosis, wound infection requiring debridement, bleeding)	7
Total	18

## Data Availability

The data presented in this study are available on request from the corresponding author due to ethical reasons.

## Supplementary Materials

The following supporting information can be downloaded at: https://www.mdpi.com/article/10.3390/jcm15187076/s1, Video S1: Case representation of Modified Grisotti surgery; Video S2: Case representation of Level 2 therapeutic mammoplasty surgery; Video S3: Case representation of Level 3 perforator flap surgery.

## Abbreviations

ACT	Adjuvant Chemotherapy
AICAP	Anterior Intercostal Artery Perforator Flap
ALND	Axillary Lymph Node Dissection
BC	Breast Cancer
BCS	Breast Conservation Surgery
BCSS	Breast Cancer-Specific Survival
BCT	Breast Conservation Therapy
CLBT	Centrally Located Breast Tumors
DCIS	Ductal Carcinoma In Situ
DFS	Disease-Free Survival
ICG	Indocyanine Green
IMC	Internal Mammary Chain
KM	Kaplan–Meier
LICAP	Lateral Intercostal Artery Perforator Flap
LMIC	Low- and Middle-Income Countries
LTAP	Lateral Thoracic Artery Perforator Flap
MDT	Multidisciplinary team
MICAP	Medial Intercostal Artery Perforator Flap
NAC	Nipple–Areola Complex
NACT/NAHT	Neoadjuvant Chemotherapy/Neoadjuvant Hormonal Therapy
NAST	Neoadjuvant Systemic Therapy
NSABP	National Surgical Adjuvant Breast Project
OBHC	Orchids Breast Health Clinic
OBS	Oncoplastic Breast Surgery
OS	Overall Survival
pCR	Pathological Complete Response
PROMS	Patient-Reported Outcome Measures
RT	Radiation Therapy/Radiotherapy
SLNB	Sentinel Lymph Node Biopsy
TM	Therapeutic Mammoplasty
TNBC	Triple-Negative Breast Cancer
