# Supplementary material for: Oncoplastic Breast Surgery Taking “Center” Stage—Managing Centrally Located Breast Tumors in Indian Cohort
Source: J Clin Med. 2026 Sep 12;15(18):7076. doi: 10.3390/jcm15187076 (PMC13607416; doi:10.3390/jcm15187076)
Supplement: Supplementary file 1 [file jcm-15-07076-s001.zip › jcm-4354214-supplementary.pdf]

Supplementary Appendix:

Supplementary Video S1: Case representation of Modified Grisotti surgery

Supplementary Video S2: Case representation of Level 2 Therapeutic Mammoplasty surgery

Supplementary Video S3: Case representation of Level 3 Perforator Flap surgery.

The videos can be found that the following Zenodo link: https://doi.org/10.5281/zenodo.18862649 (access on 4 March2026)
